# Supplementary material for: The genome sequence of Polygonum maritimum L., 1753 (Caryophyllales: Polygonaceae)
Source: Wellcome Open Res. 2026 May 29;11:321. [Version 1] doi: 10.12688/wellcomeopenres.26680.1 (PMC13310358; doi:10.12688/wellcomeopenres.26680.1)
Supplement: Peer review history

# Reviewer report: Reviewer response for version 1

**DOI:** 10.21956/wellcomeopenres.29381.r157251

**Published:** 2026-06-29

**Authors:** Lara Pereira

**Affiliations:** 1 Ecology and Evolutionary Biology, University of Sheffield, Sheffield, UK

The manuscript describes the genome of the plant *Polygonum maritimum*. The study is well designed, and the methods are robust and accurately implemented. There is a high degree of detail in the materials and methods section, ensuring reproducibility and transparency. The genome obtained, using both HiFi reads and Hi-C, is of good quality, and most of the sequence is contained in ten chromosome-level scaffolds. The authors have made an excellent job in curating the assembly and evaluating its quality post-assembly. This work contributes to better understanding of the British flora and will be useful for other scientists.

Are sufficient details of methods and materials provided to allow replication by others?

Yes

Is the rationale for creating the dataset(s) clearly described?

Yes

Are the datasets clearly presented in a useable and accessible format?

Yes

Are the protocols appropriate and is the work technically sound?

Yes

Reviewer Expertise:

Plant genetics and genomics.

I confirm that I have read this submission and believe that I have an appropriate level of expertise to confirm that it is of an acceptable scientific standard.

# Reviewer report: Reviewer response for version 1

**DOI:** 10.21956/wellcomeopenres.29381.r157252

**Published:** 2026-06-27

**Authors:** Peng-Fei Ma

**Affiliations:** 1 Kunming Institute of Botany (KIB), Chinese Academy of Sciences, Kunming, Yunnan, China

This study reports a genome assembly for the sea knotgrass ( *Polygonum maritimum*), which is now rarely found in the wild of Britain and Ireland now.  The quality of the assembled genome is generally high, with 99.95% of sequences assigned to 10 pseudo chromosomes.  In addition, the two organelle genomes were also assembled. These genomic resources will be valuable for the evolutionary study and conservation of this species.  However, the assembled haploid genome size at ~240 Mb differs sharply from the 1C-value of 520 Mb and the authors could give additional details about this difference.

Are sufficient details of methods and materials provided to allow replication by others?

Yes

Is the rationale for creating the dataset(s) clearly described?

Yes

Are the datasets clearly presented in a useable and accessible format?

Yes

Are the protocols appropriate and is the work technically sound?

Yes

Reviewer Expertise:

Plant genomics and evolution.

I confirm that I have read this submission and believe that I have an appropriate level of expertise to confirm that it is of an acceptable scientific standard.

# Reviewer report: Reviewer response for version 1

**DOI:** 10.21956/wellcomeopenres.29381.r157255

**Published:** 2026-06-17

**Authors:** Dongyan Zhao

**Affiliations:** 1 University of Florida, Gainesville, Florida, USA

"The genome sequence of Polygonum maritimum" is a well-designed project and carried out in soundly manner.

 The methods and quality control of the final assembly are well described, allowing reproducibility.

 For future work, it would be great to have genome annotation and research on its unique biological niche, e.g., its growth on sandy environment.

Are sufficient details of methods and materials provided to allow replication by others?

Yes

Is the rationale for creating the dataset(s) clearly described?

Yes

Are the datasets clearly presented in a useable and accessible format?

Yes

Are the protocols appropriate and is the work technically sound?

Yes

Reviewer Expertise:

Genome assembly, targeted genotyping platforms, QTL mapping

I confirm that I have read this submission and believe that I have an appropriate level of expertise to confirm that it is of an acceptable scientific standard.
